# Demographic, Clinical, and Surgical Risk Factors of Pars Plana Vitrectomy (PPV) for Neovascular Glaucoma (NVG) Development in Type 2 Diabetes Mellitus Patients

**DOI:** 10.7759/cureus.69571

**Published:** 2024-09-17

**Authors:** Faruk Nisic, Orhan Lepara, Rijad Jahić, Edin Begić, Lejla Alic, Almir Fajkić

**Affiliations:** 1 Ophthalmology, University Clinical Center Sarajevo, Sarajevo, BIH; 2 Human Physiology, University of Sarajevo, Sarajevo, BIH; 3 Internal Medicine/Cardiology, University Clinical Center Sarajevo, Sarajevo, BIH; 4 Cardiology, Sarajevo School of Science and Technology, Sarajevo, BIH; 5 Medical Biochemistry, University of Sarajevo, Sarajevo, BIH; 6 Pathophysiology/Internal Medicine, University of Sarajevo, Sarajevo, BIH

**Keywords:** intraocular pressure, neovascular glaucoma, pars plana vitrectomy, surgical complications, type 2 diabetes mellitus, vascular endothelial growth factor

## Abstract

Introduction: Neovascular glaucoma (NVG) is a severe type characterized by forming new blood vessels on the iris and the anterior chamber angle, often resulting from ischemic retinal diseases. Pars plana vitrectomy (PPV) is a standard surgical procedure for treating various retinal and vitreous conditions. Understanding the risk factors associated with NVG development following PPV is crucial for improving patient outcomes.

Objective: To identify and evaluate demographic, clinical, and surgical risk factors associated with developing NVG following PPV.

Patients and methods: A prospective cohort study was conducted over two years, involving 60 type 2 diabetes mellitus (T2DM) patients (31 males and 29 females; mean age 60.48±9.63 years) who underwent PPV at the Eye Clinic and Department of Clinical Immunology, University Clinical Center Sarajevo, Sarajevo, Bosnia and Herzegovina. Patients were thoroughly informed about the study, and written informed consent was obtained. Comprehensive data collection included demographic information, medical history, preoperative and postoperative eye examinations, and intraoperative details. Statistical analyses were performed using IBM SPSS Statistics for Windows, Version 21 (Released 2012; IBM Corp., Armonk, New York, United States).

Results: Within 12 months postoperatively, 17 patients (28.3%) developed NVG. Significant preoperative risk factors for NVG included prolonged duration of T2DM (p=0.037), elevated preoperative intraocular pressure (IOP) (p=0.024), and higher levels of vascular endothelial growth factor (VEGF) (p=0.011). Intraoperative factors, such as sharp dissection (p=0.000) and operative complications (p=0.004), were also significantly associated with NVG development. Multivariate logistic regression analysis identified prolonged T2DM duration (OR 1.132, p=0.023), increased preoperative IOP (OR 1.192, p=0.029), elevated VEGF levels (OR 1.002, p=0.016), and intraoperative sharp dissection (OR 0.114, p=0.006) as independent risk factors.

Conclusions: Multiple preoperative and intraoperative factors influence the development of NVG post-PPV. Prolonged T2DM duration, elevated preoperative IOP, high VEGF levels, and specific intraoperative techniques significantly increase the risk of NVG. These findings underscore the importance of careful preoperative assessment and tailored intraoperative strategies to mitigate NVG risk in PPV patients.

## Introduction

Pars plana vitrectomy (PPV) is a well-established surgical procedure utilized to address a variety of ocular conditions, including retinal detachment, vitreous and submacular hemorrhage, and traumatic eye injuries [1]. This surgical modality is particularly critical in emergent scenarios where timely intervention can prevent significant visual loss.

Although PPV is applicable across all age groups, its utilization is notably higher in the elderly population, who often present with complex and urgent conditions requiring surgical intervention. In some regions, up to 65% of PPV procedures are performed on older patients, reflecting the increased prevalence of retinal and vitreous diseases in this demographic [2]. However, the invasive nature of PPV inherently carries risks for postoperative complications, such as elevated intraocular pressure (IOP) and tractional retinal detachment (TRD), which can adversely affect patient outcomes [3].

Advancements in surgical techniques and technology have significantly improved the safety profile of PPV. Nonetheless, the demand for additional PPV procedures remains high, particularly for addressing complications following primary surgeries. Reports indicate that up to 22.8% of patients may require repeat procedures, and while severe complications like endophthalmitis and TRD have become less common, their incidence has shown minimal variation over recent decades, with rates between 4.8% and 5.5% at the one-year mark [4].

The eye is often a focal point where systemic diseases manifest, impacting ocular health and complicating surgical interventions. Conditions such as diabetes mellitus, sickle cell anemia, giant cell arteritis, and systemic lupus erythematosus can lead to significant ocular complications, which can influence the outcomes of eye surgeries. Furthermore, demographic factors like gender and age are independent risk factors that may predispose individuals to complications during and after eye surgery [2,5].

Systemic risk factors, including poorly controlled glucose levels, hypertension, and other systemic diseases, have a well-documented impact on ocular health. These factors can exacerbate existing ocular pathologies, such as elevated IOP and neovascularization, further complicating surgical outcomes [6]. Additionally, the molecular mechanisms underlying these systemic conditions often target common pathways, such as those involving vascular endothelial growth factor (VEGF), which plays a pivotal role in developing neovascular complications, particularly in type 2 diabetes mellitus (T2DM) [7].

VEGF, a key molecular target, is critically involved in the pathophysiology of numerous ocular conditions, including neovascular glaucoma (NVG). The association between VEGF and diabetes is particularly relevant in elderly populations, where VEGF-driven neovascularization contributes significantly to disease progression. Despite advances in treatment, NVG remains a major cause of severe visual impairment following vitrectomy, with reported incidence rates ranging from 2% to 18% [8].

This study aims to investigate the most prevalent risk factors related to the development of NVG before, during, and after PPV surgery. By identifying these factors, we hope to contribute to improved patient outcomes and inform strategies to mitigate the risks associated with this severe complication.

## Materials and methods

A prospective two-year cohort study was conducted at the Eye Clinic and Department of Clinical Immunology, University Clinical Center Sarajevo, Sarajevo, Bosnia and Herzegovina. The study involved 60 participants with T2DM, comprising 31 males (51.7%) and 29 females (48.3%), with a mean age of 60.48±9.63 years. All participants were thoroughly informed about the study's aims and procedures before their involvement, and written informed consent was obtained. The study was conducted following the principles of the Declaration of Helsinki and was approved by the Ethics Committee of the University Clinical Center Sarajevo (approval no. 02-07-8095).

Inclusion criteria

T2DM participants were eligible if they were male or female, aged over 18, and had undergone PPV surgery for retinal or vitreous pathology when other treatment options were not feasible during the study period.

Exclusion criteria

Participants were excluded if they had insufficient critical data, pre-existing eye conditions (e.g., glaucoma, uveitis, central retinal vein, or artery occlusion), a history of prior PPV surgery, previous intravitreal or systemic anti-VEGF treatment, a history of malignant neoplasm, or if they were unable to provide necessary data.

Data collection

A custom survey was designed to gather detailed information on each participant's demographics, including gender, age, duration of T2DM, and other relevant medical conditions. Comprehensive ophthalmic examinations were conducted, which included the measurement of best-corrected visual acuity (BCVA) using a Snellen optotype at a distance, the assessment of IOP with Goldman applanation tonometry, and a slit-lamp examination of both the anterior and posterior segments of the eye. Additionally, imaging tests, including A- and B-scan ultrasonography, were performed. Laboratory and other analyses were also conducted to measure glucose, glycated hemoglobin (HbA1c), urea, creatinine levels, and blood pressure.

Additional clinical indicators collected included the status of pan-retinal photocoagulation, lens condition, and the presence of retinal neovascularization, as well as details of retinal detachment (rhegmatogenous, traumatic, tractional), vitreous hemorrhage, and combined retinal detachments.

Intraoperative variables

Intraoperative data were meticulously documented, encompassing details on vitrectomy instrumentation, including the dimensions of the instruments used. Information on concurrent procedures, such as lens extraction and vitreous removal, was also recorded. Additionally, supplementary techniques were documented, which involved delicate tissue dissection, bleeding control through pressure increase or diathermy, intraocular laser treatment, and the use of tamponade during surgery.

Pathological microscopic analyses and data on postoperative complications, including vitreous hemorrhage within 24 hours, elevated IOP, and the onset of NVG, were collected over a 12-month follow-up period.

Surgical procedures

All surgical procedures were performed at the Eye Clinic, University Clinical Center Sarajevo, using appropriate levels of anesthesia. A Zeiss OPMI VISU 200 operating microscope (Carl Zeiss Meditec AG, Jena, Germany) was used for all surgeries, with fundus visualization achieved using the BIOM system (OCULUS GmbH, Wetzlar, Germany). Standardized surgical machines (20G Millenium or 23G Stellaris PC) were utilized according to protocol.

VEGF measurement

During the PPV surgery, a vitreous specimen was collected by inserting a 1 cc silicone tube into the vitrectomy aspiration line. The undiluted vitreous sample (approximately 0.5 to 1 cc) was centrifuged and immediately frozen at temperatures below -80°C for preservation. VEGF concentrations in the vitreous humor were measured using Quantikine Enzyme-Linked Immunosorbent Assay (ELISA) testing, with additional assessments conducted on specific postoperative days and months to monitor potential complications.

Statistical analysis

Statistical analyses were conducted using IBM SPSS Statistics for Windows, Version 21 (Released 2012; IBM Corp., Armonk, New York, United States). Data were summarized as medians with interquartile ranges (25th-75th percentiles), absolute values (N), percentages (%), and mean values±standard deviation. The normality of data distribution was assessed using the Kolmogorov-Smirnov and Shapiro-Wilk tests. Group comparisons were made using the Mann-Whitney U-test or Student’s t-test, depending on the data distribution. The Χ^2^ test and Fisher’s exact test examined dependencies between categorical variables. Univariate logistic regression analyses were performed to identify potential risk factors for developing NVG. Subsequently, a multivariate logistic regression analysis using the backward elimination method was conducted to identify statistically significant NVG risk factors, with a significance level set at p<0.05.

## Results

Table 1 presents the frequency distribution of patient characteristics, laboratory results, baseline ocular findings, surgical methods, and outcomes. In total, of 60 T2DM patients, 51.7% underwent PPV surgery alone, while 45.0% had combined PPV and phacoemulsification (PHACO) surgeries. Most patients had hypertension and normal biomicroscopic findings (73.3% and 76.7%). The mean and median values on systolic and diastolic pressure, blood glucose levels, and HbA1c were increased in the total sample. Median VEGF levels (pg/mL) were 345.0. Surgical complications were present in 31.7% of patients, while NVG was seen in 28.3% of cases in the 12th month.

**Table 1:** The frequency distribution of patient characteristics, laboratory results, baseline ocular findings, surgical methods, and outcomes The results were expressed as an absolute value (N), percentage (%), the median and interquartile range (25th-75th percentiles), and mean value±standard deviation. SBP: systolic blood pressure; DBP: diastolic blood pressure; HbA1C: glycated hemoglobin; PPV: pars plana vitrectomy; IOP: intraocular pressure; VEGF: vascular endothelial growth factor; IP: intraoperative photocoagulation; LPC: laser photocoagulation

Variables	
Hypertension	44 (73.3%)
SBP (mmHg)	170.0 (145.0-180.0)
DBP (mmHg)	90.0 (86.25-100.0)
IOP (mmHg)	16.0 (14.0-18.0)
Glucose level (mmol/L)	9.15±2.91
HbA1c (%)	8.35 (7.0-9.87)
Urea levels (μmol/L)	6.5 (5.37-8.5)
Creatinine levels (μmol/L)	84.0 (66.0-120.0)
VEGF levels (pg/mL)	345.0 (69.22-984.63)
Biomicroscope (pathological)	14 (23.3%)
PPV surgical procedure	31 (51.7%)
Operative complications	19 (31.7%)
Blunt dissection	54 (60.0%)
Sharp dissection	39 (65.0%)
IP	7 (11.7%)
Diathermy	12 (20.2%)
LPC	30 (50.0%)
Post-operative neovascular glaucoma	17 (28.3%)

During the 12-month follow-up period, 17 patients developed NVG, while 43 did not. Significant differences were observed between the groups with and without NVG; specifically, the duration of T2DM, urea levels, creatinine levels, and VEGF levels were significantly higher in the NVG group (p=0.037, p=0.022, p=0.013, p=0.011, respectively) (Table 2).

**Table 2:** Comparison of patients with and without NVG who had undergone vitrectomy The results were expressed as an absolute value (N), percentage (%), the median and interquartile range (25th-75th percentiles), and mean value±standard deviation. Group comparisons were made using the Mann-Whitney U-test or Student’s t-test, depending on the data distribution. The Χ^2^ test and Fisher’s exact test examined dependencies between categorical variables. T2DM: type 2 diabetes mellitus; SBP: systolic blood pressure; DBP: diastolic blood pressure; IOP: intraocular pressure; HbA1C: glycated hemoglobin; BCVA: best-corrected visual acuity; VEGF: vascular endothelial growth factor; PPV: pars plana vitrectomy; IP: intraoperative photocoagulation; LPC: laser photocoagulation

Variables			With NVG (n=17)	Without NVG (n=43)	p-value
Age (years)		58.05±9.15		61.44±9.75	0.223
Gender	Male	6 (35.3%)		23 (53.5%)	0.204
	Female	11 (64.7%)		20 (46.5%)	
Duration of T2DM (months)		26.0 (22.0-35.5)		22.0 (17.0-26.0)	0.037
Hypertension		12 (70.6%)		32 (74.4%)	0.762
SBP (mmHg)		160.0 (140.0-170.0)		170.0 (160.0-180.0)	0.309
DBP (mmHg)		90.0 (82.5-100.0)		90.0 (90.0-100.0)	0.933
Preoperative IOP (mmHg)		18.0 (14.5-25.5)		16.0 (14.25-18.0)	0.080
HbA1c (%)		9.0 (7.4-10.45)		7.6 (6.9-9.1)	0.086
Glucose (mmol/L)		10.04±3.22		8.78±2.73	0.134
Urea (μmol/L)		7.9 (6.45-9.1)		6.3 (4.9-8.1)	0.022
Creatinine (μmol/L)		101.0 (71.5-145.0)		76.0 (66.0-112.0)	0.013
BCVA		0.03 (0.01-0.12)		0.35 (0.10-0.50)	0.000
VEGF (pg/mL)		949. 70 (446.47-1149.18)		251.94 (48.62-819.57)	0.011
Biomicroscope (pathological)		7 (41.2%)		7 (16.3%)	0.04
Operative complications		10 (58.8%)		9 (20.9%)	0.004
Vitreous hemorrhage 1st day after PPV		7 (41.2%)		8 (18.6%)	0.069
Blunt dissection		16 (94.1%)		23 (53.5%)	0.003
Sharp dissection		14 (82.4%)		13 (30.2%)	0.000
Diathermy		5 (29.4%)		7 (16.3%)	0.225
IP		5 (29.4%)		2 (4.7%)	0.016
LPC		5 (29.4%)		25 (58.1%)	0.045

There were significant differences in categorical variables between patients with and without NVG. BCVA, pathological findings on biomicroscopy, and operative complications were notably higher in the NVG group compared to the non-NVG group (p=0.000, p=0.04, p=0.004, respectively). Additionally, intraoperative blunt dissection, sharp dissection, and intraoperative photocoagulation (IP) were more frequent in patients with NVG (p=0.003, p=0.000, p=0.016, respectively). Conversely, laser photocoagulation (LPC) was less frequent in the NVG group (p=0.049) (Table 2).

In the univariate logistic regression analysis, the following factors were identified as predictors for developing NVG after PPV surgery: longer duration of diabetes mellitus (OR 1.089, 95% CI: 1.015-1.187, p=0.020), preoperative IOP (OR 1.19, 95% CI: 1.023-1.358), preoperative BCVA (OR 1.007, 95% CI: 1.000-1.014), increased levels of VEGF (OR 1.002, 95% CI: 1.000-1.003), sharp dissection (OR 0.093, 95% CI: 0.023-0.379), pathological biomicroscopy findings (OR 0.278, 95% CI: 0.079-0.980), operative complications (OR 0.185, 95% CI: 0.055-0.624), VH on the first day after PPV (OR 5.320, 95% CI: 1.390-20.638), blunt dissection (OR 0.072, 95% CI: 0.009-0.591), IP (OR 8.542, 95% CI: 1.467-49.722), and LPC (OR 3.333, 95% CI: 0.998-11.139) (Table 3).

**Table 3:** Logistic regression analyses and the backward elimination method of NVG risk factors in subjects who underwent vitrectomy Univariate logistic regression analyses identified potential risk factors for developing NVG, followed by a multivariate regression analysis using the backward elimination method to identify statistically significant NVG risk factors at a significance level of p<0.05. T2DM: type 2 diabetes mellitus; SBP: systolic blood pressure; DBP: diastolic blood pressure; IOP: intraocular pressure; BCVA: best-corrected visual acuity; HbA1C: glycated hemoglobin; VEGF: vascular endothelial growth factor; IOP: intraocular pressure; VH: vitreous hemorrhage; IP: intraoperative photocoagulation; LPC: laser photocoagulation; PPV: pars plana vitrectomy; PHACO: phacoemulsification

		Logistic regression analyses			Backward elimination method	
Variables	Partial regression coefficient	OR (95%CI)	p-value	Partial regression coefficient	OR (95%CI)	p-value
Age (years)	-0.308	0.963 (0.906-1.023)	0.222			
Gender	0.746	2.108 (0.660-6.734)	0.208			
Duration of T2DM (months)	0.093	1.089 (1.015-1.187)	0.020	0.124	1.132 (1.018-1.260)	0.023
Hypertension	-0.129	0.825 (0.237-2.874)	0.763			
SBP (mmHg)	-0.008	0.992 (0.968-1.017)	0.526			
DBP (mmHg)	-0.007	0.993 (0.944-1.043)	0.772			
IOP (mmHg)	0.174	1.190 (1.023-1.358)	0.024	0.176	1.192 (1.019-1.395)	0.029
Preoperative BCVA	0.007	1.007 (1.000-1.014	0.039			
HbA1c (%)	0.265	1.304 (0.942-1.804)	0.110			
Glucose (mmol/L)	0.151	1.163 (0.953-1.399)	0.137			
Urea (μmol/L)	0.042	1.042 (0.898-1.210)	0.585			
Creatinine (μmol/L)	0.002	1.002 (0.993-1.011)	0.680			
Increased VEGF (pg/mL)	0.002	1.002 (1.000-1.003)	0.008	0.002	1.002 (1.000-1.003)	0.016
Biomicroscopy findings (pathological)	-1.281	0.278 (0.079-0.980)	0.046			
Operative complications (yes)	-1.686	0.185 (0.055-0.624)	0.006	-1.452	0.234 (0.055-0.992)	0.049
VH 1st day after PPV	1.671	5.320 (1.390-20.638)	0.015			
Blunt dissection (yes)	-2.663	0.072 (0.009-0.591)	0.014			
Sharp dissection (yes)	-2.337	0.093 (0.023-0.379)	0.001	-2.174	0.114 (0.024-0.534)	0.006
Diathermy (yes)	-.0762	0.467 (0.125-1.748)	0.258			
IP (yes)	2.145	8.542 (1.467-49.722)	0.017			
LPC (yes)	1.204	3.333 (0.998-11.139)	0.05			
PPV + PHACO surgery	-0.116	0.891 (0.290-2.748)	0.840			

After applying the backward elimination method of the logistic regression, the following significant preoperative risk factors for developing NVG entered the multivariable model: more prolonged duration of T2DM (OR 1.132, 95%CI 1.018-1.260, p=0.023), preoperatively increased IOP (OR 1.192, 95%CI 1.019-1.395, p=0.029), increased levels of VEGF (OR 1.002, 95%CI 1.000-1.003, p=0.002), operative complications (OR 0.234, 95%CI 0.055-0.992, p=0.049), and intraoperative sharp dissection (OR 0.114, 95%CI 0.024-0.534, p=0.006) (Table 3).

## Discussion

This study investigated the most common parameters associated with NVG development before, during, and after PPV surgery. Given the eye's sensitivity to pathological processes, timely diagnosis is crucial for preserving vision and overall ocular health.

Our study's main findings identify key preoperative risk factors for developing NVG, including prolonged duration of diabetes mellitus, elevated preoperative IOP, increased levels of VEGF, and intraoperative sharp dissection as a complication. Unlike previous studies, our research is among the first to prospectively consider perioperative risk factors influencing the development of postsurgical NVG.

NVG is a severe form characterized by forming new blood vessels obstructing aqueous humor's outflow due to posterior segment ischemia. This process leads to the development of a fibrovascular membrane on the anterior iris and iridocorneal angle, initially causing an open-angle blockage and eventually progressing to synechiae angle-closure glaucoma with elevated IOP. The neovascularization of the iris and angle typically precedes the rise in IOP [9].

Many patients with NVG exhibit significant media opacities, which complicate adequate pan-retinal photocoagulation. In such cases, PPV is crucial in managing the condition by removing media opacities, repairing the damaged posterior segment, and allowing for laser treatment via endo-photocoagulation probes [10].

NVG is most commonly associated with conditions such as central retinal vein occlusion (CRVO), proliferative diabetic retinopathy (PDR), ocular ischemic syndrome, and ocular tumors. Approximately 36% of NVG cases are linked to CRVO, 32% to PDR, and 13% to carotid artery obstruction. Given that the primary cause of NVG development is some form of retinal ischemia, it is more prevalent in elderly patients with cardiovascular risk factors such as hypertension and diabetes [11].

Increased IOP is commonly observed after PPV due to various causes, particularly in the early postoperative days. Research by Cabuk and Cekic indicates that IOP elevation occurs in up to 37% of cases following PPV [12].

However, the relationship between perioperative IOP elevation and PPV has yet to be thoroughly investigated. In similar ocular surgeries, such as trabeculectomy, Nesaratnam et al. found that perioperative IOP increases were not significantly related to postoperative complications [13].

The role of VEGF in NVG development is particularly significant. VEGF is produced in response to hypoxia and inflammation, leading to neovascularization and disease progression. VEGF, an endothelial cell-specific factor, is critical for angiogenesis and vasopermeability. Five ligands (A, B, C, D, and placenta growth factor) bind to three receptor tyrosine kinases (VEGFR-1, -2, and -3). Among these, VEGF-A is the most studied, particularly for its effects on angiogenesis and permeability, as it binds to VEGFR-1 and -2. In the retina, VEGF-A is produced by various cells, including retinal pigment epithelium, endothelial cells, pericytes, astrocytes, Müller cells, amacrine cells, and ganglion cells [14].

VEGF is a well-established risk factor for postoperative complications following PPV, especially in the context of T2DM. VEGF levels are closely related to glucose status, even in pre-diabetic states. Elevated VEGF promotes endothelial cell proliferation and increases permeability within retinal blood vessels, contributing to pathological changes such as PDR. This link suggests that elevated preoperative VEGF levels might predispose patients to NVG, particularly when combined with increased IOP in T2DM patients [7,15]. Ghanem et al. demonstrated that VEGF levels decrease following anti-VEGF treatment, supporting the relationship between VEGF, IOP, and T2DM in NVG development [16].

Lee et al. reported that the prevalence of NVG among diabetic patients in the USA is approximately 2.1% [17].

NVG is a complex disease with multiple molecular targets that require further investigation. For instance, Yu et al. analyzed the role of transforming growth factor-beta (TGF-beta) in NVG. They found elevated TGF-beta levels are significantly associated with NVG compared to the general population [18]. Similarly, Qian et al.'s two-year prospective study showed that high TGF-beta levels are linked to postoperative complications following trabeculectomy [19]. TGF-beta is a polymorphic cytokine implicated in the pathogenesis of T2DM, with its activity influenced by genetic polymorphisms and pathological triggers [20].

TGF beta is a very polymorphic cytokine whose role is also proven in the pathogenesis of T2DM. Therefore, its activity depends on genetic polymorphism besides some pathological activators, and its levels are strongly related to the deterioration of systemic diseases such as T2DM and consequential implications on NVG representing directions for new research projects due to anti-VEGF treatment in terms of dose titration which provides better efficacy of anti-VEGF treatment [14,15].

Additionally, long-term follow-up studies are needed to investigate the persistence and recurrence of NVG beyond the 12-month period post-surgery. Exploring preoperative anti-VEGF treatments and their effectiveness in reducing NVG risk in diverse populations could yield valuable insights for clinical practice. Especially because of VEGF action in the proliferation of endothelial cells further causing the obliteration of blood vessels. Comparative studies focusing on different surgical techniques and their impact on NVG development, particularly regarding sharp dissection and intraocular manipulation, could help establish best practices. Therefore, it could be intriguing to provide further data about different forms of anti-VEGF therapy such as pills or eye drops in order to compare data between different usage forms. Finally, research into molecular markers like TGF-beta and the role of glycemic control in modulating NVG risk will be crucial in developing more personalized treatment approaches.

Despite the significant findings, several limitations must be acknowledged. First, the sample size of 60 participants, while adequate for preliminary analysis, may not provide sufficient statistical power to generalize the findings to broader populations. Larger cohort studies are necessary to validate our results and further explore the nuances of NVG development post-PPV surgery. Second, the study was conducted at a single clinical center, which may introduce location-specific biases in patient demographics and clinical practices. Multi-center studies would help mitigate this limitation and provide a more comprehensive understanding of NVG risk factors.

Furthermore, our study did not account for all potential risk factors. For example, we did not measure systemic inflammatory markers, which could further explain the role of chronic inflammation in NVG development. Moreover, we did not explore genetic predispositions or molecular markers like TGF-beta, which have been associated with NVG in other studies. Including these in future studies would provide a more detailed understanding of the molecular mechanisms behind NVG.

Another limitation is the relatively short follow-up period of 12 months, which may not capture the long-term progression of NVG and its complications. Extended follow-up studies would be necessary to determine whether NVG persists or worsens over time. Last, patient adherence to pre- and postoperative treatment protocols (e.g., anti-VEGF injections) was not systematically monitored, which could affect the outcomes.

Finally, the observational nature of our study precludes establishing causation between the identified risk factors and NVG development. However, this study gives a perspective for further research work regarding NVG and its potential in the field of molecular targets and the pathophysiology of NVG through molecular targets related to VEGF. This study includes many variables potentially leading to a statistical challenge for logistic regression, but we have abrogated that risk with the backward selection method.

## Conclusions

Postoperative complications can aggravate recovery after the PPV procedure, whereas NVG represents the most severe one. Risk factors associated with NVG development include system T2DM and preoperatively increased IOP and VEGF levels. Therefore, specific measures must be considered to decrease preoperative risk factors. Those measures might include decreasing preoperative IOP and anti-VEGF treatment before PPV surgery and minimizing intraoperative manipulation like sharp dissection.
